# Linear and Angular Deviations of Complete-Arch Digital Implant Impressions Using Three Scan Body Systems: An In Vitro Study

**DOI:** 10.3390/dj14100686

**Published:** 2026-10-21

**Authors:** Igor Blivajs, Igor Smojver, Yuval Reiser, Stella Jurić, Ena Hadžović, Ivica Pelivan, Dragana Gabrić

**Affiliations:** 1 Department of Otolaryngology, Head and Neck Surgery, University Hospital Centre Zagreb, Kišpatićeva 12, HR-10000 Zagreb, Croatia; igor.blivajs@kbc-zagreb.hr; 2 Department of Oral Surgery, School of Dental Medicine, University of Zagreb, HR-10000 Zagreb, Croatia; dgabric@sfzg.hr; 3 School of Dental Medicine, University of Zagreb, HR-10000 Zagreb, Croatia; yreiser@sfzg.hr (Y.R.); sjuric@sfzg.hr (S.J.); ehadzovic@sfzg.hr (E.H.); 4 Department of Removable Prosthodontics, School of Dental Medicine, University of Zagreb, HR-10000 Zagreb, Croatia; pelivan@sfzg.unizg.hr; 5 Clinical Hospital Center Zagreb, University Dental Clinic, HR-10000 Zagreb, Croatia

**Keywords:** complete-arch digital impression, dental implants, scan body, linear deviation, angular deviation, in vitro study

## Abstract

**Background:** Scan body systems may affect the transfer of implant positions in complete-arch digital impressions. This in vitro study compared linear and angular deviations among three systems in a mandibular All-on-4 model. **Methods:** Ten repeated scans per system (exploratory sample) were acquired with a TRIOS 5 intraoral scanner for Straumann Titanium, Straumann Exact and TruAbutment ioConnect scan bodies. A laboratory-scanned cast from a conventional impression served as the reference; deviations are relative to this workflow. After conversion to a common geometry and complete-arch registration, linear deviations were measured in a standardized 2D cross-section and angular deviations between corresponding component axes. Scan-level means of four sites were compared using Welch’s ANOVA and Games–Howell tests (α = 0.05). **Results:** Mean linear deviations were 0.187 ± 0.025 mm (Exact), 0.234 ± 0.005 mm (Titanium) and 0.135 ± 0.060 mm (TruAbutment; *p* < 0.001). Titanium exceeded Exact (*p* < 0.001) and TruAbutment (*p* = 0.0014); Exact and TruAbutment did not differ significantly (*p* = 0.060). Mean angular deviations were 1.304 ± 0.067°, 2.691 ± 0.139° and 1.380 ± 0.065° (*p* < 0.001); Titanium exceeded both systems (*p* < 0.001), but Exact–TruAbutment did not (*p* = 0.050). **Conclusions:** Within this model and reference workflow, Titanium showed greater linear and angular deviations than Exact and TruAbutment. No difference was established between Exact and TruAbutment. The findings do not demonstrate equivalence or clinically acceptable fit.

## 1. Introduction

The rehabilitation of edentulous patients with implant-supported fixed prostheses has become a predictable and widely adopted treatment modality in contemporary dentistry. The All-on-4 treatment concept, which utilizes four strategically positioned implants to support a full-arch prosthesis, has demonstrated excellent long-term clinical outcomes [1]. This approach offers patients immediate function, reduced treatment complexity, and favorable biomechanical load distribution while avoiding extensive bone augmentation procedures [2]. However, the clinical success of full-arch implant-supported rehabilitations depends critically on the passive fit of the prosthetic framework, which in turn relies on the dimensional accuracy of the impression technique used to capture the three-dimensional positions and angulations of the implant abutments [3,4].

The transition from conventional elastomeric impression techniques to digital workflows has fundamentally transformed implant prosthodontics over the past decade. Digital impression systems offer numerous advantages, including enhanced patient comfort, elimination of dimensional changes associated with impression material setting and stone expansion, immediate visualization of captured data, and seamless integration with computer-aided design and manufacturing (CAD/CAM) workflows [3,5]. Recent systematic reviews and meta-analyses have demonstrated that digital impressions achieve accuracy comparable to conventional impressions in most clinical situations, particularly for single implants and partially edentulous cases [3,4]. However, the evidence regarding digital impression accuracy for complete-arch implant rehabilitations remains inconsistent and controversial. A 2026 meta-analysis of 37 studies found no significant overall difference in accuracy between digital and conventional impressions, although conventional methods were slightly favored in cases involving more than four implants [3]. The accuracy of intraoral scanners appears to decline as the scanning span increases, with cumulative errors potentially compromising the passive fit of full-arch frameworks [4,6].

Multiple factors have been identified as potential sources of error in digital implant impressions, including scanner technology and generation, scanning pattern and strategy, operator experience, implant depth and angulation, span length between implants, and notably, scan body (also termed scan abutment or implant scan body) design [7,8]. Scan bodies serve as the critical interface between the physical implant position and the digital representation captured by the intraoral scanner [9]. These components vary considerably in their geometric design, height, diameter, material composition (titanium, polyetheretherketone), surface characteristics, and retention mechanisms (screw-retained versus friction-fit) [7,10]. A 2023 systematic review examining the influence of scan body design on intraoral scanning accuracy identified 20 studies investigating various design parameters yet concluded that the existing evidence does not enable concrete conclusions regarding optimal scan body design or whether specific scan body characteristics interact with scanner technologies [7]. Recent investigations have demonstrated that scan body height, geometry, and material significantly influence digital impression accuracy, with shorter scan bodies and specific geometric configurations yielding superior trueness in some studies [6,10].

The clinical significance of impression accuracy in full-arch implant rehabilitations extends beyond technical precision to encompass biological and mechanical outcomes. Passive fit of the prosthetic framework is considered critical for long-term success, as the absence of periodontal ligaments around dental implants limits adaptation compared to natural teeth [10]. Framework misfits have been identified as risk factors for various mechanical and biological complications that may compromise long-term implant therapy success, including screw loosening and fracture, ceramic veneer fracture, and marginal bone loss [11,12]. Intraoral scanners rely on best-fit algorithms that require continuous reference points for three-dimensional image reconstruction, a challenge that is particularly pronounced in completely edentulous arches where limited anatomical landmarks are available.

Despite the growing body of literature on digital impression accuracy, significant knowledge gaps remain regarding the comparative performance of different scan body designs and acquisition methods in the specific clinical context of edentulous All-on-4 rehabilitations. The majority of existing studies have evaluated either partially edentulous scenarios or have not systematically compared multiple scan body designs using standardized measurement protocols. Understanding how scan body systems influence both linear deviation (the positional discrepancy of corresponding implant components) and angular deviation (the discrepancy in component axis orientation) is essential for optimizing digital workflows and ensuring predictable prosthetic outcomes in full-arch implant cases. Linear accuracy is critical for achieving proper framework fit and avoiding cantilever effects, while angular accuracy directly impacts screw access channel positioning, emergence profile esthetics, and biomechanical load distribution.

This in vitro study compared linear and angular deviations among three scan body systems used with the same intraoral scanner in a mandibular All-on-4 model. The null hypothesis was that the mean deviations relative to the reference dataset would not differ among systems for either outcome.

## 2. Materials and Methods

### 2.1. Study Design and Ethical Considerations

This in vitro comparative study evaluated linear and angular deviations of digital impressions obtained with three scan body systems and one intraoral scanner in a mandibular All-on-4 model.

The study was conducted in a controlled laboratory environment at the University of Zagreb, School of Dental Medicine. The study was approved by the Ethics Committee of the Faculty of Dentistry in Zagreb (CLASS 003-01/26-05/05, URBROJ: 251-60-4/119-8, dated 13 May 2026).

### 2.2. Fabrication of the Master Model

A commercial acrylic mandibular model (Institut Straumann AG, Basel, Switzerland) simulating a completely edentulous arch served as the standardized reference for all measurements. Four implant analogs (Straumann Bone Level Tapered, RC, Ø 4.1 mm, length 10 mm; Institut Straumann AG, Basel, Switzerland) were embedded in the model according to the All-on 4 configuration: two axial anterior implants positioned in the lateral incisor regions and two posterior implants tilted at 30° positioned in the second premolar regions. The implant positions were predetermined using coDiagnostiX software (version 10.9; Dental Wings Inc., Montreal, QC, Canada) to ensure precise placement and reproducibility.

Straumann Screw-Retained (SR) abutments were connected to all four implant analogs and tightened to 35 Ncm using a calibrated torque wrench (Straumann Torque Wrench, Institut Straumann AG), following the manufacturer’s recommendations. The model was inspected visually and tactilely to confirm stable abutment seating and absence of mobility. This master model served as the physical reference for all subsequent digital acquisitions.

### 2.3. Preparation of the Reference Dataset (REF)

**Figure 1:**
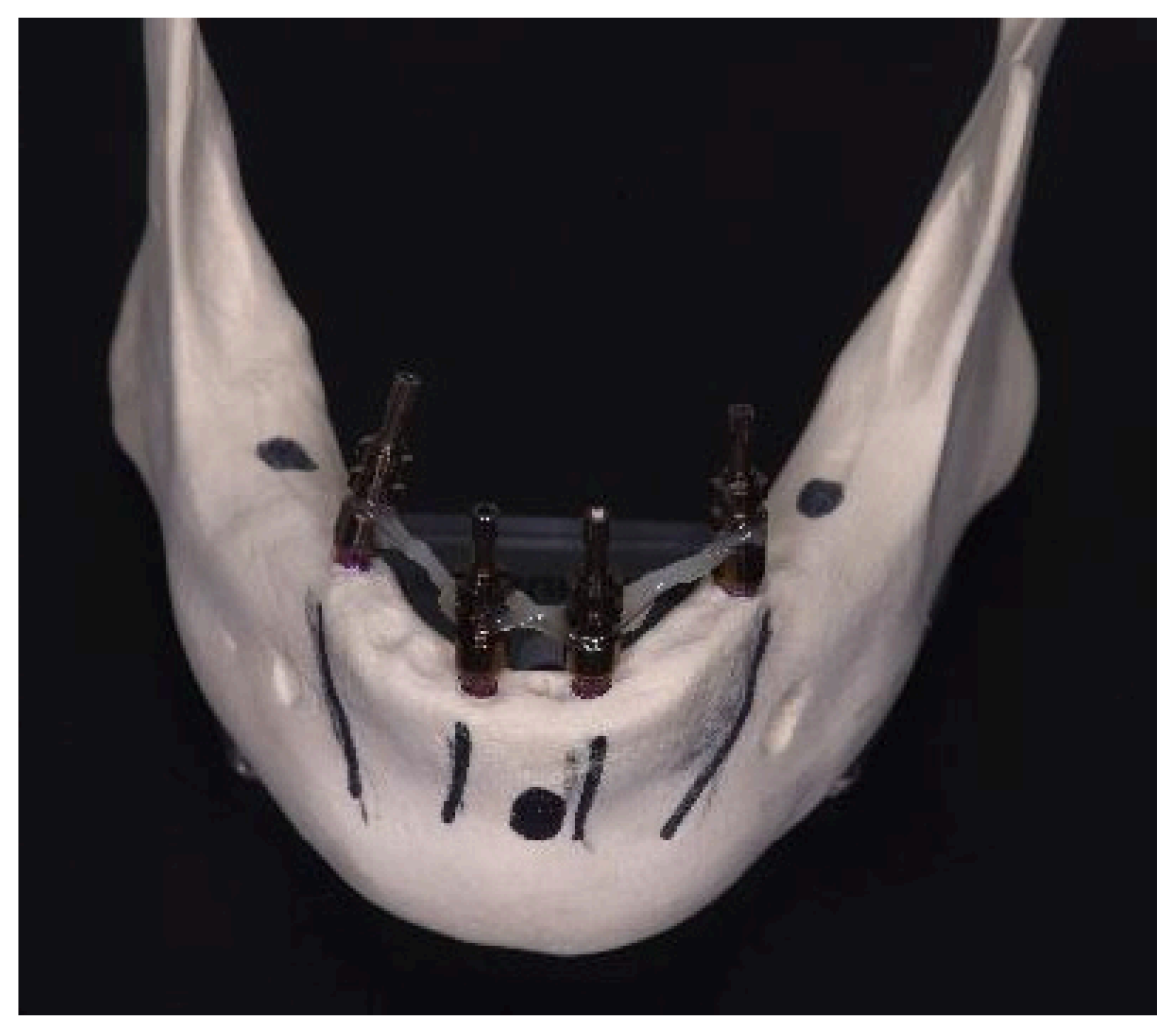
Prior to impression-making, open tray impression copings were attached to the abutments and rigidly splinted with orthodontic wire and light curing composite to prevent micro movement during impression removal and to maximize dimensional stability.

Conventional open-tray impression of the master model was obtained using polyether impression material (Impregum Penta, 3M ESPE, Seefeld, Germany) in a custom acrylic tray. Prior to impression-making, open-tray impression copings were attached to the SR abutments and rigidly splinted using 0.7 mm diameter stainless steel orthodontic wire and a light-polymerizing flowable composite resin (Tetric N-Flow, shade A2; Ivoclar Vivadent, Schaan, Liechtenstein) to prevent micromovement during impression removal and ensure maximum dimensional stability (Figure 1). The splinting material was light-cured for 40 s per segment using an LED curing unit (Bluephase PowerCure; Ivoclar Vivadent, Schaan, Liechtenstein) with a light intensity of 1200 mW/cm^2^. After complete polymerization of the impression material (according to manufacturer specifications), the impression was carefully removed, inspected for defects, and poured within 30 min using Type IV dental stone (Fujirock EP; GC Corporation, Tokyo, Japan) mixed according to the manufacturer instructions.

**Figure 2:**
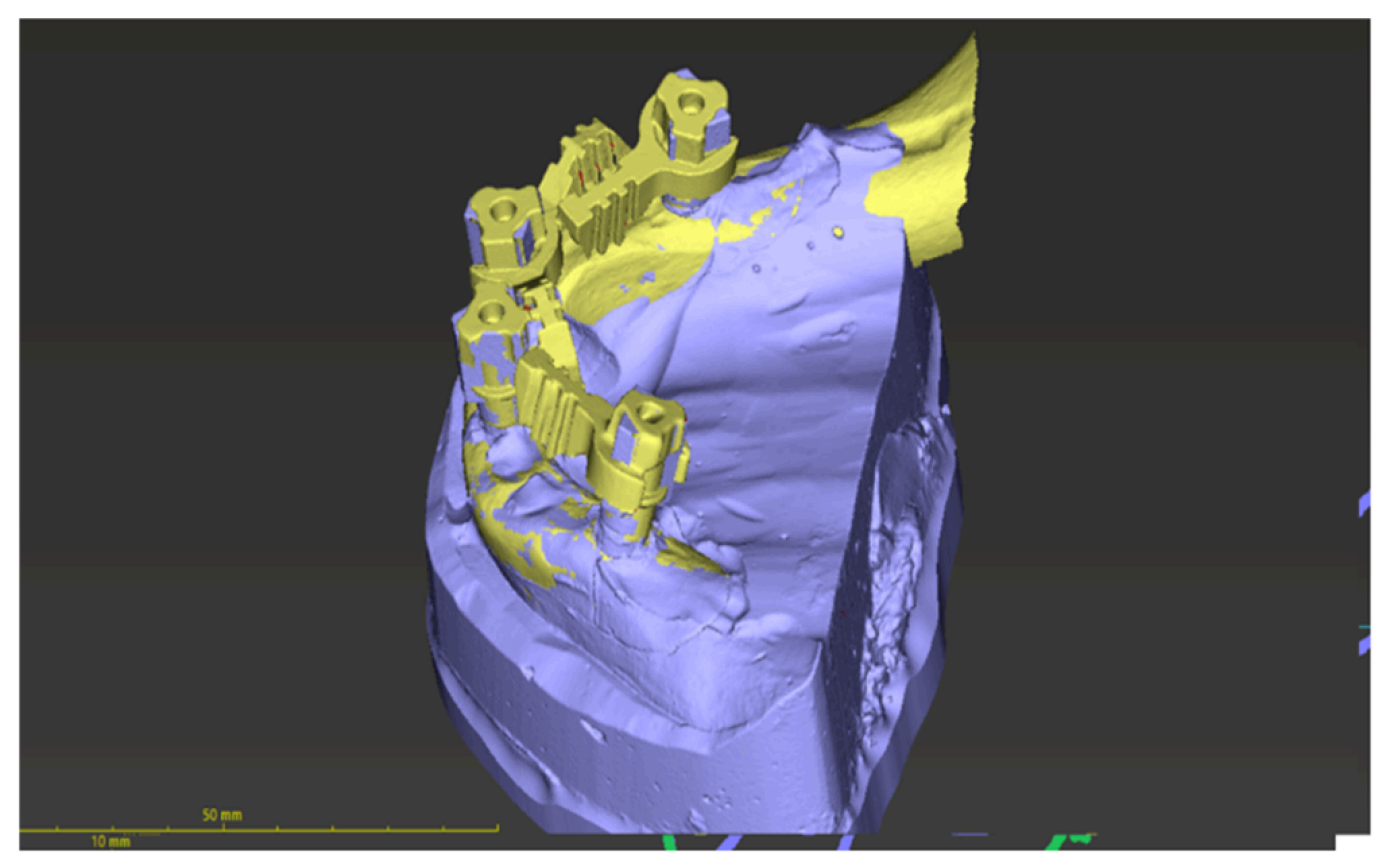
Three-dimensional reference model (STL) obtained by laboratory digitization of the conventionally fabricated stone cast. The dataset served as the reference for all subsequent deviation measurements. The reference model (purple) and the experimental dataset (yellow) are shown before measurement.

After complete setting of the stone (minimum 45 min), the working cast was separated from the impression. The same type of titanium scan bodies as those used in the Titanium experimental group were used to digitize the reference cast. The cast was scanned with a 3Shape E4 laboratory scanner (3Shape A/S, Copenhagen, Denmark). The resulting STL file was designated as the reference dataset (REF) for comparison with the experimental complete-arch scans (Figure 2). Calibration of the laboratory scanner does not validate the dimensional transfer from the acrylic master model to the stone cast. Independent metrological characterization of the master model and repeated reference acquisitions were not performed. Accordingly, the reference dataset represents a conventional reference workflow comprising impression transfer, cast fabrication, scan-body seating, and laboratory digitization, rather than an independently characterized metrological ground truth. All reported deviations are therefore relative to the tested reference workflow and should not be interpreted as absolute errors relative to the physical master model.

### 2.4. Experimental Groups and Sample Size

**Figure 3:**
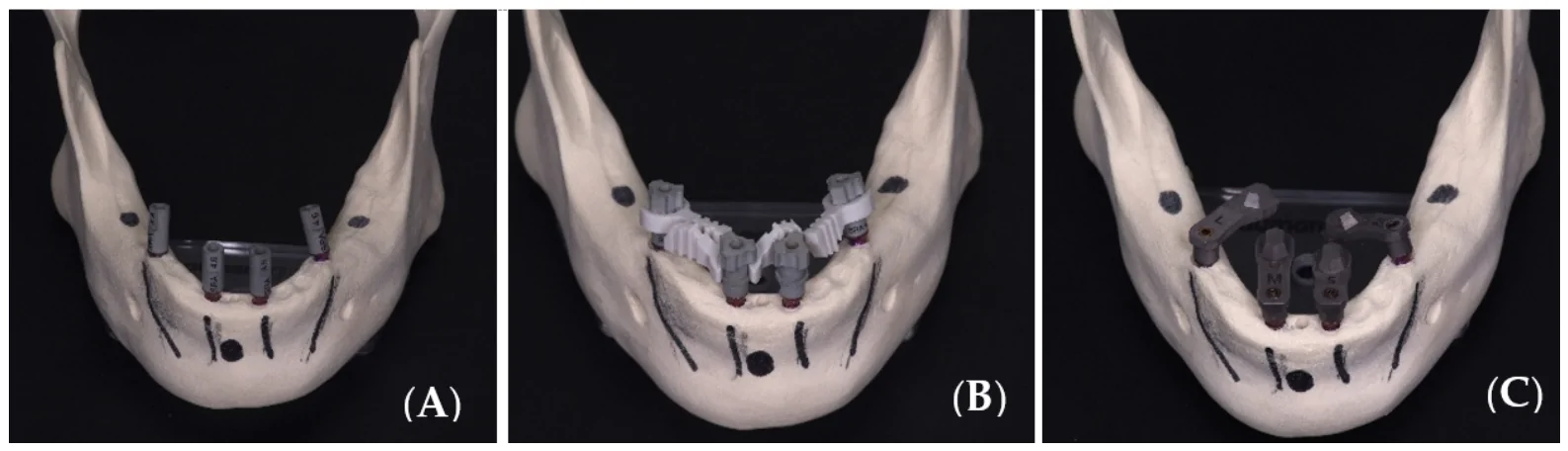
Experimental groups (**A**–**C**) showing the different scan body designs used with the intraoral scanner: (**A**) titanium scan body, (**B**) exact scan body, (**C**) TruAbutment ioConnect scan body.

The sample was divided into three experimental groups based on the scan body system:

- **Group A** (Figure 3A): 3Shape TRIOS 5 intraoral scanner + Straumann titanium scan body (Institut Straumann AG, Basel, Switzerland)
- **Group B** (Figure 3B): 3Shape TRIOS 5 intraoral scanner + Straumann EXACT scan body system (Scanbody BASE SRA 4.6 for edentulous IOS, REF 025.0084, with Short LINK, REF 025.0087V4; Institut Straumann AG, Basel, Switzerland)
- **Group C** (Figure 3C): 3Shape TRIOS 5 intraoral scanner + TruAbutment ioConnect (TruAbutment ioConnect scan body, TruAbutment Inc., Irvine, CA, USA)

Ten repeated full-arch scans were obtained for each scan body system. Each scan yielded four site-specific measurements per outcome, recorded in the following order: posterior right (FDI 45), anterior right (FDI 42), anterior left (FDI 32), and posterior left (FDI 35). Thus, 40 implant-level measurements per system were available for each outcome. The four linear deviations within each scan were averaged to obtain one full-arch linear value, and the four angular deviations were averaged separately to obtain one full-arch angular value. The analysis therefore included 10 scan-level observations per system for each outcome.

Ten repeated complete-arch acquisitions were obtained for each scan body system, yielding 30 scan-level observations in total. The number of acquisitions was determined by feasibility rather than by an a priori sample-size calculation. Accordingly, the study was considered exploratory, and the sample size was not intended to establish equivalence or to ensure adequate precision for all pairwise comparisons. The tested scan body systems and their respective processing workflows are detailed in Supplementary Table S1.

### 2.5. Digital Scanning Protocol

All digital scans were performed by a single calibrated operator to eliminate inter-operator variability. The operator completed a standardization session consisting of five practice scans with each system prior to data collection to ensure familiarity with the scanning protocols and minimize learning curve effects.

Environmental Standardization: All scans were performed under controlled environmental conditions to minimize potential confounding variables. Room temperature was maintained at 20 °C ± 1 °C, and ambient lighting was standardized at 500 lux using overhead LED fixtures with no external daylight. Although relative humidity was not specifically measured, all scans were performed consecutively under identical environmental conditions to minimize variability.

For Groups A, B, and C, digital impressions were acquired using the 3Shape TRIOS 5 wireless intraoral scanner (3Shape A/S, Copenhagen, Denmark) with firmware version 23.1. The TRIOS 5 system employs ultrafast optical sectioning technology with confocal microscopy and structured light projection, capturing up to 3000 3D images per second.

The appropriate scan bodies for each group were attached to the SR abutments on the master model and tightened to a torque of 15 Ncm. One initially new set of four scan bodies was used for each system, and each component was returned to its original site for every repetition. Before each scan, the scan bodies were inspected to ensure complete seating and absence of debris. The scanning sequence followed the manufacturer’s recommended protocol for edentulous arches:

1. Initial capture of the first scan body (posterior right implant);
2. Continuous scanning motion moving anteriorly along the right side;
3. Crossing to the left anterior region;
4. Continuing posteriorly along the left side;
5. Final capture of remaining scan bodies with multiple overlapping passes.

Each scan was completed in a single continuous sequence without interruption. The operator maintained approximately 5–15 mm between the scanner tip and the scan bodies, with a scanning speed of approximately 20 mm/s, consistent with manufacturer recommendations. Each scanning procedure was repeated 10 times per group (*n* = 10). Between repetitions, the scan bodies were removed, cleaned with 70% isopropyl alcohol, reattached to their original sites and tightened to 15 Ncm. No sterilization was performed. The ten analyzed scans were acquired in a fixed block order (Titanium, Exact, TruAbutment); acquisition order was neither randomized nor interleaved. Within-system variability therefore includes any effect of repeated component seating in addition to optical acquisition and subsequent processing.

All STL files were exported directly from the TRIOS software (version: 3Shape Unite 23.1) in native resolution without compression or smoothing filters applied.

### 2.6. Deviation Analysis: Linear and Angular Deviations

Deviations of the experimental datasets relative to the reference dataset were assessed after conversion of the scanned scan bodies to a common component geometry. For the Titanium and Exact systems, as well as for the titanium scan bodies used to digitize the reference cast, the scanned scan bodies were aligned with the corresponding scan-body library models in the 3Shape software (version: 3Shape Dental System23.1). This procedure represented each implant site using the library geometry of the Straumann scan body for the SRA abutment. For the TruAbutment system, the scanned flag geometry was processed in the ioConnect software (version: TruSuite v1.0.29.), where the operator selected the center of each hexagon. The software calculated the corresponding abutment positions and exported them using the same Straumann SRA scan-body geometry. Because all implant sites carried Straumann SRA abutments, corresponding sites in all datasets were represented by geometrically identical components before measurement. However, the conversion procedures were system-specific and were not validated against datasets with known spatial transformations; their contribution therefore remains part of the tested workflow.

Initial correspondence between each experimental dataset and the reference dataset was established using four fixed model landmarks, comprising three externally marked locations and one lingual indentation. This was followed by software-based alignment of the complete datasets, with rigid transformations limited to rotation and translation; no scaling was applied. Registration was performed separately in 3Shape Dental Designer for linear measurements and in coDiagnostiX for angular measurements. In 3Shape Dental Designer, complete-dataset best-fit alignment was performed following landmark-based initialization. In coDiagnostiX, point-based matching was followed by software fine alignment. The resulting alignment was reviewed by the operator. Each complete-arch dataset was transformed as a single unit, preserving the relative positions and orientations of its four implant sites; no individual component was independently repositioned to improve agreement with the reference. The component surfaces were not excluded from the final complete-dataset alignment, and independence of the final fit from the measured component surfaces was therefore not established. The outcomes represent residual deviations following the applied registration rather than errors measured in an independently established coordinate system.

#### 2.6.1. Linear Deviation

**Figure 4:**
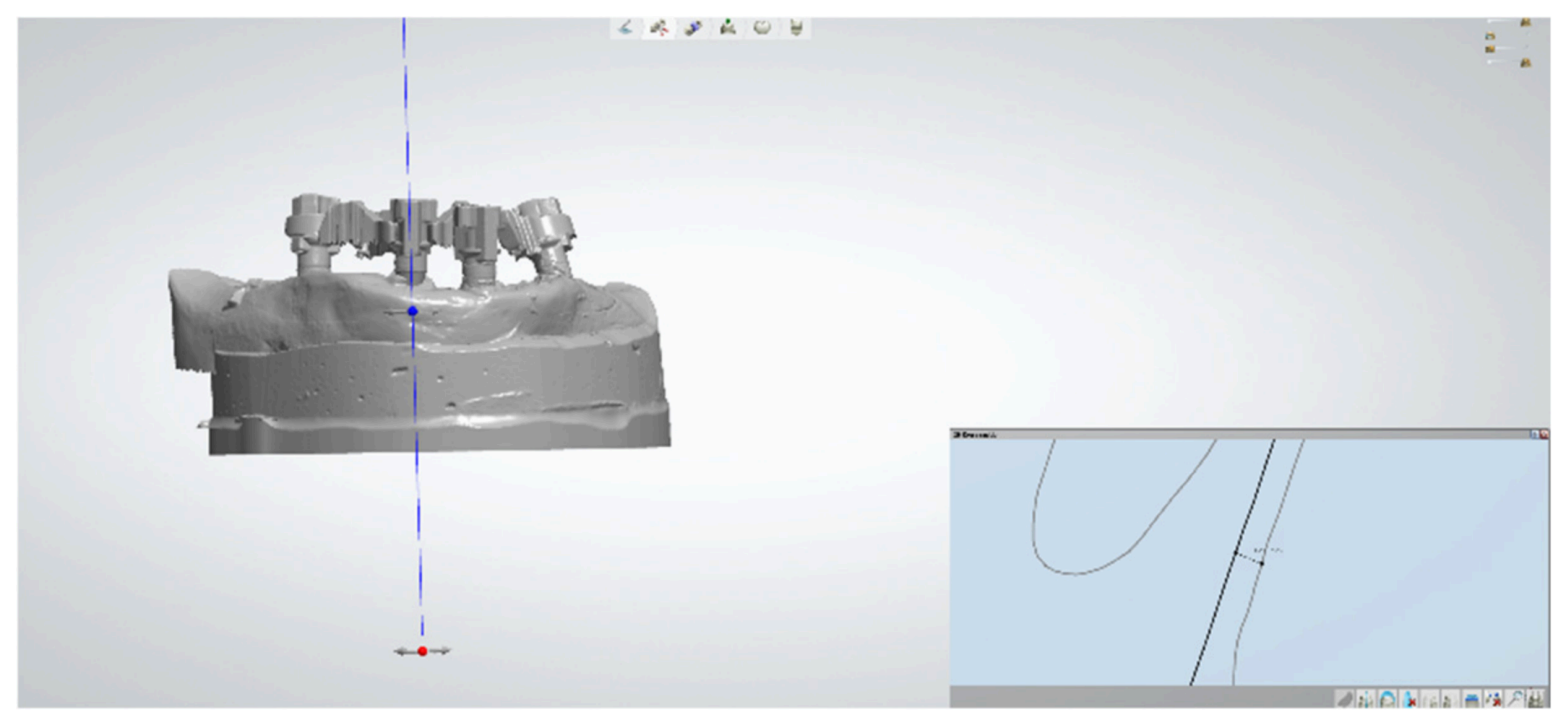
Linear deviation measurement in 3Shape Dental Designer after conversion to the common component geometry and complete-arch alignment. The operator placed measurement points on corresponding component edges or surfaces within a standardized 2D cross-section of consistent height and orientation, and the software calculated the distance in millimeters. The four site distances were averaged to obtain one value per scan.

Linear deviations were evaluated in 3Shape Dental Designer (version 2023; 3Shape A/S, Copenhagen, Denmark). Following complete-arch alignment, linear deviations were measured using the software’s two-dimensional (2D) cross-section tool. The section height and orientation were kept consistent across the compared datasets. The operator manually placed measurement points on corresponding component edges or surfaces within the selected cross-section, and the software calculated the distance between these points in millimeters. Because all datasets had been converted to the same component geometry, the selected edges or surfaces belonged to geometrically identical components. Four distances were recorded per scan, in the sequence FDI 45, 42, 32 and 35. Their arithmetic mean represented the linear deviation for that complete-arch scan. This procedure was repeated for all ten acquisitions per system. The measured endpoint represents a cross-sectional linear discrepancy and does not represent a three-dimensional centroid displacement or displacement of the implant-platform center (Figure 4).

#### 2.6.2. Angular Deviation

Angular deviations were evaluated in coDiagnostiX (version 10.9; Dental Wings Inc., Montreal, QC, Canada). The converted experimental and reference datasets were exported as STL files and imported into coDiagnostiX as model scans. Each experimental dataset was registered to the reference dataset using point-based initial alignment on the four fixed model landmarks, followed by the software’s fine-alignment procedure. The congruence of the complete models was checked visually in all views. No further alignment was applied during the subsequent angular measurements.

**Figure 5:**
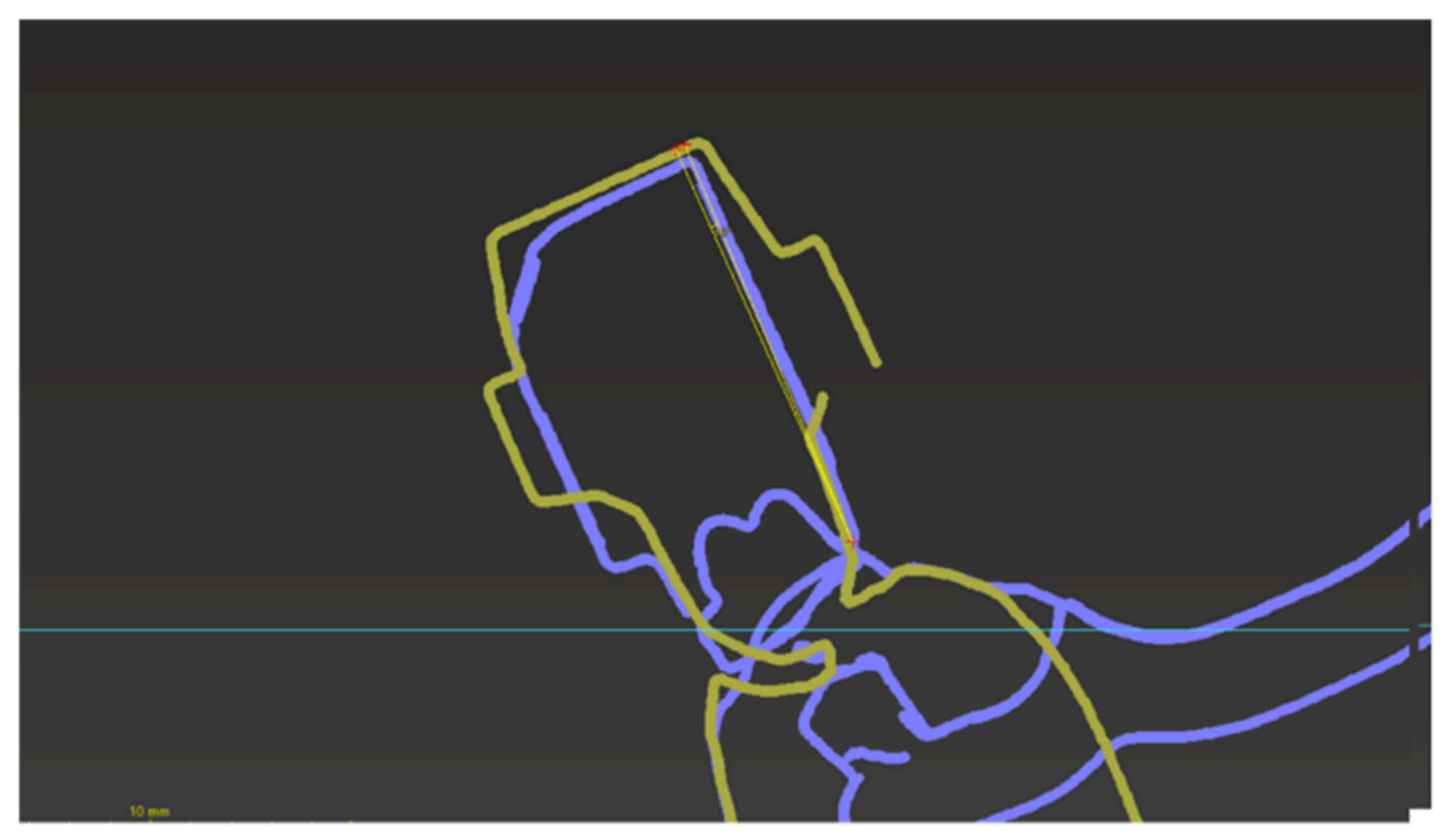
Angular deviation measurement in coDiagnostiX following complete-arch registration. The angle between the longitudinal axes of corresponding components in the experimental and reference datasets was measured at each of the four implant sites. The four site-specific angles were averaged to obtain one angular deviation value per scan.

Because coDiagnostiX does not provide scan-body library matching or automatic axis extraction for imported model scans, angles were measured manually using the software’s angle tool, which calculates the angle between two lines defined by three points. At each implant site, the operator placed a common vertex and one additional point along the longitudinal axis of each corresponding experimental and reference component. The software then calculated the angle between the two axes. Four angles were recorded per scan, in the sequence FDI 45, 42, 32 and 35. Their arithmetic mean represented the angular deviation for that complete-arch scan. This procedure was repeated for all ten acquisitions per system. Angular deviations were expressed in degrees (Figure 5).

### 2.7. Statistical Analysis

Statistical analyses were performed using Python 3.12.14 with SciPy 1.17.0. Descriptive statistics were calculated from the full-arch means and included the mean and sample standard deviation, median with the 25th and 75th percentiles, range, and a two-sided 95% t confidence interval for the group mean. Normality was assessed using the Shapiro–Wilk test. Homogeneity of variances was evaluated using the median-centered Levene test (Brown–Forsythe modification).

Because variances differed between systems for both outcomes, group means were compared using Welch’s one-way analysis of variance. Games–Howell comparisons were used to compare all three pairs within each outcome, with adjusted *p*-values and simultaneous 95% confidence intervals for the mean differences. The significance level was 0.05. No additional adjustment was applied across the two outcome families, so outcome-specific inference was considered exploratory. Descriptive eta-squared was calculated as SSbetween/SStotal and was reported separately from the Welch test statistic. Calculations used unrounded values; no observations were excluded.

The scan was the unit of analysis. The three system groups were analyzed as separate groups of repeated acquisitions. Matching measurement numbers across systems were not treated as evidence of pairing. The 40 implant-level values per system were not analyzed as 40 independent scans.

The sample size of ten acquisitions per system was not determined by an a priori calculation; therefore, statistical inference was considered exploratory. Retrospective power estimates based on the observed effects are presented in Supplementary Table S2 for transparency only and were not used to establish sample adequacy or equivalence. Additional exploratory analyses included site-specific descriptive statistics and assessment of the association between consecutive scan index and deviation within each system using Spearman rank correlation, with Holm adjustment for the six comparisons across the two outcomes and three systems (Supplementary Tables S3 and S4). These analyses were performed in Python using NumPy version 2.3.5 and SciPy version 1.17.0.

## 3. Results

The analysis included ten complete-arch scan-level observations per system for each outcome. Each scan-level value was calculated as the arithmetic mean of four implant-site measurements. Shapiro–Wilk tests did not indicate significant departures from normality in any group (Table 1 and Table 2). However, variance homogeneity was rejected for both linear and angular deviations using the median-centered Levene test (Table 3). Accordingly, between-system comparisons were performed using Welch’s analysis of variance, followed by Games–Howell post hoc comparisons. Given the exploratory sample and the limitations of the reference and measurement workflows, the findings should be interpreted as differences in measured deviations relative to the tested reference workflow.

### 3.1. Linear Deviation

**Figure 6:**
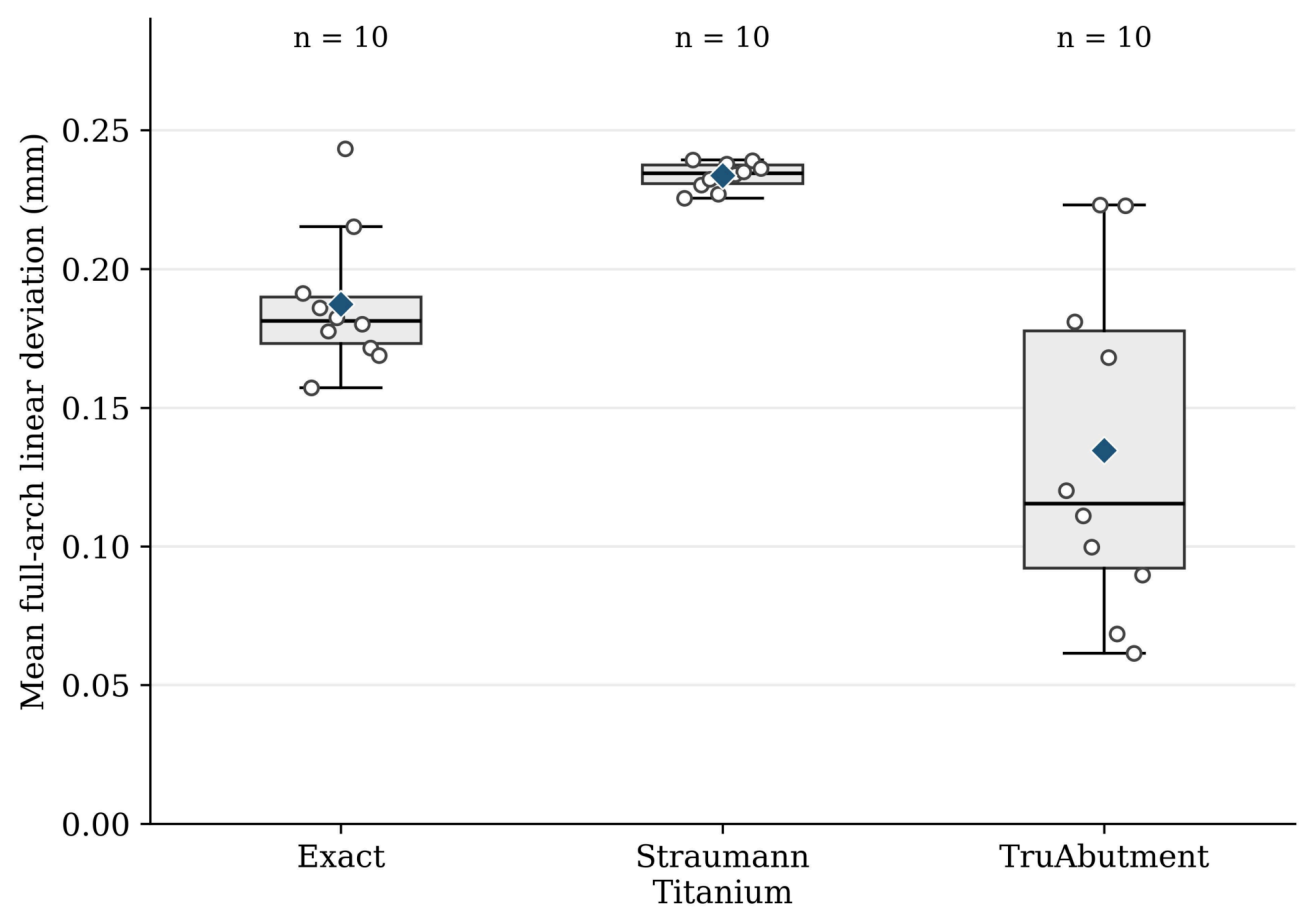
Distribution of mean full-arch linear deviations across scan body systems. Each open circle represents one scan-level mean derived from four implant-level measurements (*n* = 10 per system). Boxes show the interquartile range, horizontal lines indicate medians, and whiskers extend to the most extreme observations within 1.5 interquartile ranges of the box. Blue diamonds indicate group means. Horizontal point offsets are used only to improve visibility.

TruAbutment had the lowest mean linear deviation (0.135 ± 0.060 mm), followed by Exact (0.187 ± 0.025 mm) and Titanium (0.234 ± 0.005 mm). The corresponding 95% confidence intervals for the means were 0.092–0.178 mm, 0.170–0.205 mm, and 0.230–0.237 mm, respectively (Table 1; Figure 6).

Welch’s analysis of variance detected an overall difference among systems, F(2, 12.507) = 28.136, *p* = 2.347 × 10^−5^. Descriptive eta-squared was 0.562. Games–Howell comparisons showed greater deviation for Titanium than for Exact (mean difference 0.04628 mm; simultaneous 95% CI 0.02414–0.06841 mm; *p* < 0.001) and TruAbutment (0.09908 mm; 95% CI 0.04593–0.15222 mm; *p* = 0.0014) (Table 4). The difference between Exact and TruAbutment was not significant (Exact minus TruAbutment, 0.05280 mm; 95% CI −0.00212 to 0.10772 mm; *p* = 0.05990).

### 3.2. Angular Deviation

**Figure 7:**
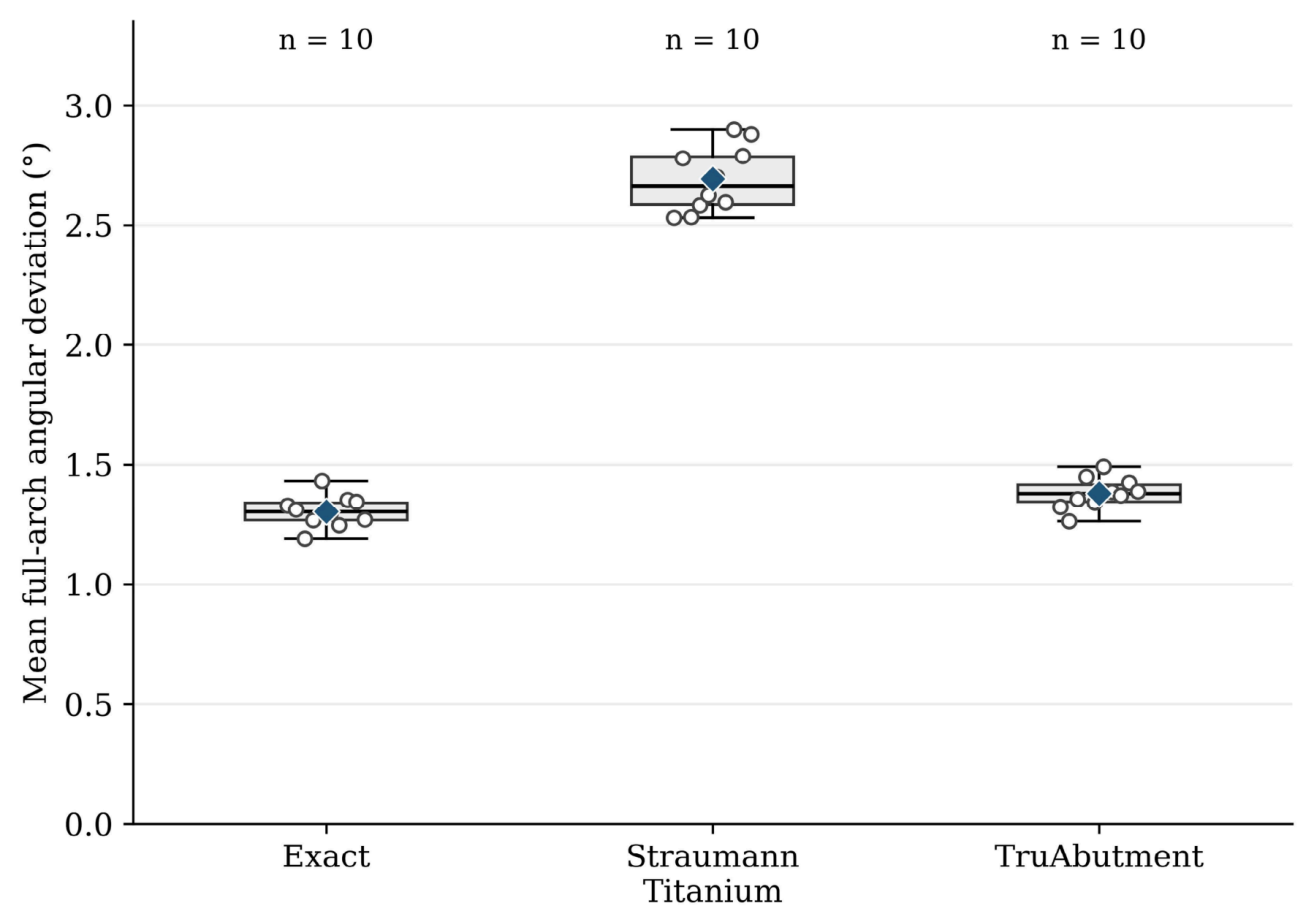
Distribution of mean full-arch angular deviations across scan body systems. Each open circle represents one scan-level mean derived from four implant-level measurements (*n* = 10 per system). Boxes show the interquartile range, horizontal lines indicate medians, and whiskers extend to the most extreme observations within 1.5 interquartile ranges of the box. Blue diamonds indicate group means. Horizontal point offsets are used only to improve visibility.

Exact had the lowest mean angular deviation (1.304 ± 0.067°), followed by TruAbutment (1.380 ± 0.065°) and Titanium (2.691 ± 0.139°). The corresponding 95% confidence intervals for the means were 1.257–1.352°, 1.333–1.426°, and 2.592–2.791°, respectively (Table 2; Figure 7).

Welch’s analysis of variance detected an overall difference among systems, F(2, 16.974) = 411.968, *p* = 4.118 × 10^−15^. Descriptive eta-squared was 0.980. Titanium showed greater deviation than both Exact (mean difference 1.38705°; simultaneous 95% CI 1.25852–1.51558°) and TruAbutment (1.31175°; 95% CI 1.18354–1.43996°); both adjusted *p*-values were below 0.001. The Exact–TruAbutment comparison did not reach the 0.05 significance threshold (Exact minus TruAbutment, −0.07530°; 95% CI −0.15068 to 0.00008°; *p* = 0.05026). This result does not establish equivalence between these systems.

### 3.3. Site-Level and Acquisition-Order Analyses

Site-specific descriptive results are presented in Supplementary Table S3. Mean linear deviations at the tilted posterior sites (FDI 45 and 35) were descriptively higher than those at the axial anterior sites (FDI 42 and 32) for Exact (0.215 versus 0.160 mm) and TruAbutment (0.165 versus 0.105 mm), but not for Titanium (0.233 versus 0.234 mm). No consistent anterior–posterior pattern was observed for angular deviation.

The scan-level standard deviation for Titanium’s linear outcome was 0.00483 mm, whereas the descriptive standard deviation across its 40 site-level measurements was 0.01190 mm, with site-specific standard deviations ranging from 0.00868 to 0.01652 mm. The corresponding scan-level standard deviations were 0.02493 mm for Exact and 0.06014 mm for TruAbutment. These differences illustrate that variability in the average scan-level outcome does not fully represent variability at individual implant sites.

In the exploratory scan-index analysis (Supplementary Table S4), Titanium’s angular deviation was positively associated with consecutive scan number before multiplicity adjustment (Spearman’s ρ = 0.745; unadjusted *p* = 0.0133), but this association did not remain statistically significant after Holm adjustment (adjusted *p* = 0.0800). No other association was statistically significant after adjustment. These analyses are exploratory and do not establish acquisition-order effects, progressive arch distortion or the reproducibility of reconstructed three-dimensional implant positions.

## 4. Discussion

The evaluated scan body systems differed in their mean linear and angular deviations relative to the reference dataset. Titanium showed greater deviations than Exact and TruAbutment for both outcomes. The comparisons between Exact and TruAbutment did not reach statistical significance after Games–Howell adjustment. The results describe the performance of the tested systems within one acquisition setup; they do not isolate the contribution of any individual geometric feature.

### 4.1. Linear Deviation

The evaluated systems differed in their mean deviations relative to the reference workflow. Titanium showed greater linear deviation than Exact and TruAbutment. Although TruAbutment had the lowest observed mean, its difference from Exact did not reach statistical significance after Games–Howell adjustment (*p* = 0.05990). The larger dispersion in the TruAbutment group should be considered when interpreting its lower mean. These findings do not support a claim that all three systems differed from one another.

Previous studies have reported that scan body design can affect the trueness of full-arch digital implant impressions [13]. Meneghetti et al. [6] reported that different scan body designs produced 3D deviations ranging from 72.27 μm to over 120 μm when scanning six implants in a maxillary model, with scan body geometry being a significant determinant of accuracy. Similarly, Alkindi et al. [14] found that short scan bodies achieved significantly better trueness (37.1 to 51.9 μm) compared to long scan bodies (89.6 to 127.9 μm) in angulated implant scenarios. Because the present study compared complete commercial systems, the observed differences cannot be attributed to any individual geometric or material feature.

Scan body geometry and surface features have been proposed as factors influencing optical recognition and linear and angular deviations [15,16,17]. In the present study, however, the systems differed simultaneously in geometry, material, surface characteristics, manufacturing tolerance, library recognition and software conversion, and they were acquired in fixed blocks. The observed differences therefore characterize the tested system–workflow combinations and do not identify the mechanism responsible.

The small linear SD of the Titanium group refers to the scan-level mean of four sites. Position-specific SDs were larger (0.009–0.017 mm), and similar distance magnitudes can arise from deviations in different spatial directions. The low dispersion of this averaged scalar should therefore not be interpreted as established reproducibility of reconstructed implant positions. Descriptively, linear deviations of Exact and TruAbutment were larger at the tilted posterior sites than at the axial anterior sites. Because positions within an arch are dependent and the study was not powered for site comparisons, this pattern was not tested formally and should be examined in studies designed for position-specific analysis.

### 4.2. Angular Deviation

Titanium showed greater angular deviation than Exact and TruAbutment. The Exact–TruAbutment comparison yielded an adjusted *p*-value of 0.05026 and a confidence interval that included zero. The data therefore did not establish a difference at α = 0.05, but the result should not be interpreted as evidence of equivalence. Its proximity to the decision threshold also supports reporting the confidence interval and avoiding categorical claims of similar performance.

In the Titanium group, angular deviation showed an increasing trend with consecutive scan index before multiplicity adjustment (Spearman ρ = 0.745; Holm-adjusted *p* = 0.080). Drift related to repeated seating or acquisition time therefore cannot be excluded, and the fixed acquisition blocks prevent separation of system and order effects. Linear and angular deviations should also not be regarded as independent error sources, because an angular discrepancy can shift component edges within the measured cross-section.

Angular accuracy is a critical parameter in full-arch implant rehabilitation, as rotational errors can significantly affect the fit of screw-retained prostheses even when linear positional accuracy appears acceptable. Previous study proposed angular deviation thresholds in the context of complete-arch implant prostheses [18]; however, these thresholds are not directly validated by the present experimental design. Therefore, the angular deviations observed in this study should be interpreted as measurements of positional transfer relative to the reference workflow rather than as direct indicators of clinical acceptability or prosthetic fit.

### 4.3. Comparison with Conventional Impressions and Other Digital Systems

A recent systematic review and meta-analysis by Alfaraj et al. [3] comparing digital and conventional impressions for complete-arch implant-supported prostheses found no significant overall difference in accuracy between the two techniques. However, subgroup analysis revealed that conventional methods were slightly favored in cases with more than four implants, whereas photogrammetry-based systems produced slightly greater accuracy than conventional impressions [3].

These findings suggest that the accuracy of digital impressions may be influenced not only by scan body design but also by the number of implants and the specific digital acquisition technology employed.

### 4.4. Clinical Implications

The measured deviations describe positional transfer within the tested laboratory workflow. They do not establish clinically acceptable prosthetic fit because framework fabrication and passivity were not evaluated. The cross-sectional linear deviations are not direct measurements of the marginal implant–framework gap or of displacement at the implant platform. Angular means of 1.304° and 1.380° also cannot be described as meeting a 1° threshold. No clinical acceptability threshold was validated by this experiment.

### 4.5. Factors Affecting Accuracy of Full-Arch Digital Impressions

Beyond scan body design, multiple factors have been shown to influence the accuracy of intraoral scanning for edentulous arches. A systematic review by Carneiro Pereira et al. [15] identified several critical variables, including the type of intraoral scanner, scanning technique, environmental conditions (temperature, humidity, ambient lighting), inter-implant distance and angulation, and scan body material. Polyether ether ketone (PEEK) and titanium are currently the most commonly used scan body materials. Owing to its favorable optical properties and reduced light reflectivity, PEEK may facilitate more reliable optical acquisition during intraoral scanning, whereas titanium provides superior mechanical stability and resistance to deformation [19]. However, titanium’s higher reflectivity may increase optical acquisition challenges, while repeated sterilization and excessive tightening torque may adversely affect the dimensional stability of polymer scan bodies over time. Current evidence suggests that neither material demonstrates universal superiority, and scanning accuracy depends on the interaction between scan body material, design, intraoral scanner technology, and clinical scanning conditions [20]. Because material was not isolated as an experimental factor, the present results cannot be attributed to scan body material. In the present study, environmental conditions were standardized (500 lux ambient lighting, controlled temperature), and all scans were performed by a single calibrated operator to minimize variability. However, the influence of scanning pattern was not specifically evaluated.

Gómez-Polo et al. [21] demonstrated that scanning pattern significantly affects accuracy, with circumferential scanning patterns achieving lower root mean square (RMS) error values compared to zigzag patterns for complete-arch implant scans. Additionally, mandibular arches consistently produced lower RMS errors compared to maxillary arches, regardless of scanning pattern [21]. The present study utilized a mandibular model, which may have contributed to relatively favorable outcomes compared to what might be expected in maxillary applications.

Alternative full-arch transfer strategies include stereophotogrammetry and reference-assisted workflows. Pozzi et al. [18] prospectively compared intraoral optical scanning with stereophotogrammetry for complete-arch implant impressions in vivo. Pellegrino et al. [22] described an Impression Reference-assisted approach that maintains a spatial reference across the pre-surgical prosthetic project, intraoperative digital acquisition, dataset matching and immediate prosthetic transfer. The latter was a feasibility case series and did not quantify implant positional or registration accuracy. These approaches address related transfer challenges but were not evaluated in the present experiment.

### 4.6. Limitations

This study has several limitations. First, it evaluated repeated scans of a single mandibular All-on-4 model using one intraoral scanner and one operator. The results therefore do not establish the variability that would occur across independently fabricated models, operators or clinical cases. Laboratory scanning also does not reproduce clinical influences such as saliva, patient movement or soft-tissue interference. Second, the reference dataset was derived from one conventional impression, one stone cast and one laboratory scan. The master model was not independently characterized using metrology traceable to a national metrology institute, and repeated reference acquisitions were not performed. The reference therefore represents a conventional reference workflow rather than an independently established metrological ground truth. Because the reference cast was digitized using the same scan-body type as the Titanium group, a systematic bias toward or against that system cannot be excluded. Third, the conversion of the scan-body systems to a common component geometry involved system-specific procedures that were not validated against known spatial transformations. Any error introduced during library alignment or hexagon-based position detection may therefore have contributed to the measured outcomes. Registration involved landmark-based initialization followed by software best-fit alignment of the complete datasets, performed separately for the linear and angular evaluations. Independence of the final alignment from the component surfaces used for measurement was not established. The reported values should consequently be interpreted as residual deviations after the applied registration, rather than absolute errors in an independently defined coordinate system. Fourth, linear deviations were measured between manually selected points in standardized two-dimensional cross-sections. This method does not capture out-of-plane discrepancies and does not quantify three-dimensional implant-platform displacement or prosthetic framework fit. Angular deviations were also measured manually, using a three-point angle tool; the results may therefore depend on the operator’s placement of the measurement points. Repeatability of these measurements on unchanged datasets and inter-examiner reproducibility were not assessed. Fifth, the scan bodies were removed and reseated between acquisitions. Although a standardized tightening torque of 15 Ncm was used and component seating was checked, variability related to repeated seating could not be separated from optical acquisition and subsequent processing. The systems were acquired in fixed blocks rather than in randomized or interleaved order, so system effects cannot be fully separated from acquisition-order effects. The exploratory scan-index analysis does not eliminate this uncertainty. Sixth, the four implant-site measurements were averaged to obtain one scan-level outcome. This approach avoids treating measurements from the same scan as independent replicates, but averaging may obscure site-specific differences, particularly between axial anterior and tilted posterior implants. The study was not designed or powered for formal comparisons between implant positions, and the scalar outcomes do not indicate the spatial direction of the discrepancies. Finally, the sample size was not determined by an a priori calculation, limiting the precision of between-system estimates, particularly for comparisons involving Exact and TruAbutment. The study did not isolate individual system characteristics, such as material or geometry, as independent experimental factors; potential scan-body wear was not quantified; and prosthetic framework fit and passivity were not evaluated. No formal equivalence analysis was performed. The findings should therefore not be interpreted as evidence of clinical acceptability or equivalence between systems.

### 4.7. Future Research Directions

Future investigations should include in vivo clinical studies comparing the accuracy of different scan body designs under actual clinical conditions. Long-term clinical studies evaluating the relationship between digital impression accuracy and prosthetic complications (screw loosening, framework fracture, biological complications) would help establish evidence-based thresholds for clinically acceptable accuracy. Finally, investigation of the interaction between scan body design and different intraoral scanner technologies would help clinicians select optimal combinations for specific clinical scenarios.

## 5. Conclusions

Within the tested model and reference workflow, the Straumann Titanium system showed greater mean linear and angular deviations than the Exact and TruAbutment systems. Differences between Exact and TruAbutment were not statistically established for either outcome. These findings compare complete system-workflow combinations and should be interpreted in light of the exploratory sample, reference limitations, and measurement procedures. The reported values represent residual deviations after complete-arch best-fit registration, not absolute positional or angular errors in an independently defined coordinate system. The study does not establish clinical acceptability or equivalence between the evaluated systems.

## Figures and Tables

**Table 1:** Descriptive statistics of full-arch linear deviation (mm).

System	Mean ± SD	Median (Q1, Q3)	Min–Max	95% CI of Mean	Shapiro–Wilk *p*
Exact	0.187 ± 0.025	0.181 (0.173, 0.190)	0.157–0.243	0.170–0.205	0.117
Straumann Titanium	0.234 ± 0.005	0.235 (0.231, 0.237)	0.226–0.239	0.230–0.237	0.463
TruAbutment	0.135 ± 0.060	0.116 (0.092, 0.178)	0.062–0.223	0.092–0.178	0.253

*n* = 10 scans per system. Each scan-level value is the mean of four site-specific measurements. Q1 and Q3 denote the 25th and 75th percentiles calculated by linear interpolation. CI, confidence interval; SD, standard deviation. Group mean confidence intervals use the t distribution with 9 degrees of freedom. Values are rounded for display; all tests used unrounded values.

**Table 2:** Descriptive statistics of full-arch angular deviation (°).

System	Mean ± SD	Median (Q1, Q3)	Min–Max	95% CI of Mean	Shapiro–Wilk *p*
Exact	1.304 ± 0.067	1.304 (1.268, 1.341)	1.190–1.432	1.257–1.352	0.983
Straumann Titanium	2.691 ± 0.139	2.662 (2.586, 2.787)	2.531–2.900	2.592–2.791	0.259
TruAbutment	1.380 ± 0.065	1.380 (1.345, 1.416)	1.263–1.492	1.333–1.426	0.997

Short system labels are used for readability. “Straumann Scanbody” in the angular source file corresponds to Straumann Titanium; “Tru Abutment” corresponds to TruAbutment.

**Table 3:** Variance homogeneity and overall group comparisons.

Outcome	Median-Centered Levene F(2,27); *p*	Welch F (df1, df2)	Welch *p*	η^2^
Linear	8.930; 0.001055	28.136 (2, 12.507)	2.347 × 10^−5^	0.562
Angular	5.998; 0.006994	411.968 (2, 16.974)	4.118 × 10^−15^	0.980

The variance test is the median-centered Levene test (Brown–Forsythe modification). Welch ANOVA is used for overall comparisons. η^2^ = SSbetween/SStotal is a descriptive effect-size measure reported separately from the Welch test. df, degrees of freedom.

**Table 4:** Games–Howell pairwise comparisons.

Outcome	Comparison (First–Second)	Mean Difference	Simultaneous 95% CI	Adjusted *p*
Linear	Exact–Straumann Titanium	−0.04628	−0.06841 to −0.02414	<0.001
Linear	Exact–TruAbutment	0.05280	−0.00212 to 0.10772	0.05990
Linear	Straumann Titanium–TruAbutment	0.09908	0.04593 to 0.15222	0.0014
Angular	Exact–Straumann Titanium	−1.38705	−1.51558 to −1.25852	<0.001
Angular	Exact–TruAbutment	−0.07530	−0.15068 to 0.00008	0.05026
Angular	Straumann Titanium–TruAbutment	1.31175	1.18354 to 1.43996	<0.001

Mean differences are first system minus second system, in mm for linear deviation and degrees for angular deviation. Confidence intervals are simultaneous 95% Games–Howell intervals for the three comparisons within each outcome. Negative values indicate lower deviation in the first system. No additional adjustment was applied across the two outcomes.

## Data Availability

The original contributions presented in this study are included in the article/Supplementary Material. Further inquiries can be directed to the corresponding author.

## Supplementary Materials

The following supporting information can be downloaded at: https://www.mdpi.com/article/10.3390/dj14100686/s1, Table S1: Scan body systems and processing workflows; Table S2: Retrospective power analysis; Table S3: Position-specific descriptive results; Table S4: Exploratory scan-index associations.

## Abbreviations

The following abbreviations are used in this manuscript:

CAD/CAM	Computer-aided design and manufacturing
SR	Screw retained
STL	Standard tessellation language
REF	Reference dataset
PEEK	Polyether ether ketone
