# Supplementary material for: Linear and Angular Deviations of Complete-Arch Digital Implant Impressions Using Three Scan Body Systems: An In Vitro Study
Source: Dent J (Basel). 2026 Oct 21;14(10):686. doi: 10.3390/dj14100686 (PMC13702890; doi:10.3390/dj14100686)
Supplement: Supplementary file 1 [file dentistry-14-00686-s001.zip › dentistry-4631839-supplementary.pdf]

# Supplementary Materials

## Linear and Angular Deviations of Complete-Arch Digital Implant Impressions Using Three Scan Body Systems: An In Vitro Study

**Table S1.** Scan body systems and processing workflows.

| Feature | Titanium | Exact | TruAbutment |
|---|---|---|---|
| Manufacturer | Institut Straumann AG | Institut Straumann AG | TruAbutment Inc. |
| System | Straumann metal scan body for SRA, Ø 4.6 (REF 025.0081) | Straumann EXACT: Scanbody BASE SRA 4.6 for edentulous IOS (titanium; REF 025.0084) with Short LINK for Scanbody BASE (POM; REF 025.0087V4) | TruAbutment ioConnect |
| Abutment level | Straumann SRA | Straumann SRA | Straumann SRA |
| Common output geometry | Straumann SRA scan body (library) | Straumann SRA scan body (library) | Straumann SRA scan body (library) |
| Scanner | TRIOS 5 (firmware 23.1) | TRIOS 5 (firmware 23.1) | TRIOS 5 (firmware 23.1) |
| Physical set | 4 initially new bodies; reused at fixed sites | 4 initially new bodies; reused at fixed sites | 4 initially new bodies; reused at fixed sites |
| Applied torque | 15 Ncm | 15 Ncm | 15 Ncm |
| Analyzed acquisitions | 10; first block | 10; second block | 10; third block |
| Sterilization | None | None | None |
| Conversion to common geometry | Library alignment in 3Shape software | Library alignment in 3Shape software | Hexagon-based position detection in ioConnect software; export as Straumann SRA scan body geometry |

SRA, Straumann screw-retained abutment; IOS, intraoral scanning; POM, polyoxymethylene. The applied torque is an experimental condition and is not a claim that one torque is the manufacturer's recommendation for every component. All systems were converted to the same Straumann SRA scan body geometry before linear measurement.

**Table S2.** Retrospective power analysis.

| Outcome | Descriptive $\eta^2$ | Cohen f | Conventional ANOVA power | Welch simulation |
|---|---|---|---|---|
| Linear | 0.56166 | 1.13195 | 0.99979 | 99,999 / 100,000 |
| Angular | 0.97975 | 6.95557 | > 0.99999 | 100,000 / 100,000 |

Values are based on the observed effects and are descriptive only. $f = \sqrt{[\eta^2/(1 - \eta^2)]}$; $\alpha = 0.05$; three groups of ten acquisitions; $\lambda = 30f^2$. Welch simulation: 100,000 normal-data replicates using the observed group means and standard deviations (seed 20261009). Post hoc power based on observed effects does not justify the sample size, establish equivalence or describe the power of individual Games–Howell contrasts. For a conventional omnibus sensitivity calculation, N = 30 gives 80% power at approximately f = 0.600.

**Table S3.** Position-specific descriptive results.

| Outcome | System | FDI 45 | FDI 42 | FDI 32 | FDI 35 |
|---|---|---|---|---|---|
| Linear (mm) | Exact | 0.2203 ± 0.0692 | 0.1832 ± 0.0721 | 0.1358 ± 0.0718 | 0.2101 ± 0.0815 |
| Linear (mm) | Titanium | 0.2316 ± 0.0165 | 0.2308 ± 0.0119 | 0.2373 ± 0.0098 | 0.2348 ± 0.0087 |
| Linear (mm) | TruAbutment | 0.1655 ± 0.1125 | 0.1105 ± 0.1075 | 0.0987 ± 0.1220 | 0.1635 ± 0.1031 |
| Angular (°) | Exact | 1.2753 ± 0.1170 | 1.2849 ± 0.1110 | 1.3359 ± 0.1448 | 1.3211 ± 0.1014 |
| Angular (°) | Titanium | 2.7827 ± 0.2434 | 2.6796 ± 0.2539 | 2.7265 ± 0.2449 | 2.5766 ± 0.2099 |
| Angular (°) | TruAbutment | 1.4335 ± 0.1428 | 1.3901 ± 0.1187 | 1.3339 ± 0.0860 | 1.3609 ± 0.1122 |

mean ± SD across ten acquisitions. FDI, Fédération Dentaire Internationale tooth notation; SD, standard deviation. Positions within an acquisition are dependent; no test treats the 40 site values as independent scans. Descriptive patterns are not evidence of a position effect or of progressive arch distortion.

**Table S4.** Exploratory scan-index associations.

| Outcome | System | Spearman ϱ | Unadjusted p | Holm-adjusted p |
|---|---|---|---|---|
| Linear | Exact | −0.188 | 0.6032 | 1.0000 |
| Linear | Titanium | 0.430 | 0.2145 | 0.8580 |
| Linear | TruAbutment | −0.394 | 0.2600 | 0.8580 |
| Angular | Exact | 0.115 | 0.7514 | 1.0000 |
| Angular | Titanium | 0.745 | 0.0133 | 0.0800 |
| Angular | TruAbutment | 0.588 | 0.0739 | 0.3694 |

Two-sided Spearman rank correlation tests between deviation and consecutive scan index within each system, with Holm adjustment over six exploratory checks (two outcomes × three systems). Adjusted non-significance does not exclude drift. Because systems were acquired in fixed blocks (Titanium, then Exact, then TruAbutment), system effects cannot be separated from between-block time effects.
